# Computational Simulation Studies on the Binding Selectivity of 1-(1*H*-Benzimidazol-5-yl)-5-aminopyrazoles in Complexes with FGFR1 and FGFR4

**DOI:** 10.3390/molecules23040767

**Published:** 2018-03-01

**Authors:** You-Lu Pan, Yan-Ling Liu, Jian-Zhong Chen

**Affiliations:** College of Pharmaceutical Sciences, Zhejiang University, Hangzhou 310058, China; 11319001@zju.edu.cn (Y.-L.P.); liuyanling610@zju.edu.cn (Y.-L.L.)

**Keywords:** FGFR1, FGFR4, inhibitors, selectivity, MD-simulation, binding free energy

## Abstract

Fibroblast growth factor receptor 1 (FGFR1) has become a potential target for the treatment of cancer. Designing FGFR1-selective inhibitors remains fundamental to the development of anti-cancer drugs because of highly sequential homology among FGFR subtypes. In present work, four inhibitors were examined with intermolecular interaction patterns with FGFR1 and FGFR4, respectively, for the exploration of binding mechanisms by applying a combined approach of computational techniques, including flexible docking, binding site analyses, electronic structure computations, molecular dynamic simulations, and binding free energy predictions. Molecular simulation-predicted binding conformations and pharmacophoric features of these molecules in the active pocket of either FGFR1 or FGFR4. MMPB(GB)SA-calculated binding free energies were accordant with the ordering of their tested potency values. Furthermore, in silico mutations of two residues (FGFR1: Tyr563 and Ser565) were also performed to check their impact on ligand binding by applying MD simulations and binding free energy calculations. The present studies may provide a structural understanding of the FGFR1-selective mechanism. The viewpoints from computational simulations would be valuable guidelines for the development of novel FGFR1-selective inhibitors.

## 1. Introduction

As a kind of receptor tyrosine kinases (RTKs), the fibroblast growth factor receptor (FGFR) has exhibited potential as a target for the development of anti-tumor drugs. FGFR has been studied intensely because of its clinical significance [1,2]. There are at least four highly sequential homology FGFR subtypes: FGFR1, FGFR2, FGFR3, and FGFR4 [3]. In comparison with other RTKs [4,5], these four FGFRs are transmembrane proteins containing an extracellular ligand binding domain, a single transmembrane helix, and a cytosolic region with tyrosine kinase activity [6]. By cross-phosphorylating tyrosine residues at the dimer state bonded to FGFs, FGFRs trigger cellular signaling pathways, like RAS-MAPK, PI3K-AKT, and PKC [7,8], initiating distinct downstream signals cascades to control a wide range of biological functions [8,9]. However, dysregulation and over-activation of FGF-FGFRs may lead to many malignancies, including breast cancer [10], lung cancer [11], and gastric cancer [12]. The kinase domain of FGFR was believed to be an underlying therapeutic target for the development of corresponding drugs to inhibit phosphorylation [13,14,15].

The kinase region of FGFR consists of two high-conserved domains, a small N-terminus domain and a large C-terminus domain, connected by a flexible hinge region [16,17,18]. An ATP-binding site is located in the cleft between these two subdomains and hinge region [19]. A ‘DFG motif’ was defined to have different conformations in the active (DFG-in mode) vs. inactive (DFG-out mode) state of the kinase region. The phenyl side-chain of the ‘DFG motif’ is in a conformation compatible with ATP binding in ‘DFG-in’ mode. The kinase may be regulated by the activated loop region next to the hinge region when the ‘DFG motif’ has its conformation transition from “on” state (active conformation) to the “off” state (inactive conformation) [17,20]. All reported co-crystal structures of inhibitor-FGFR complexes showed the ‘DFG-in’ conformation of the kinase domain, meaning the inhibitors preferred the ‘DFG-in’ mode [20].

**Figure 1:**
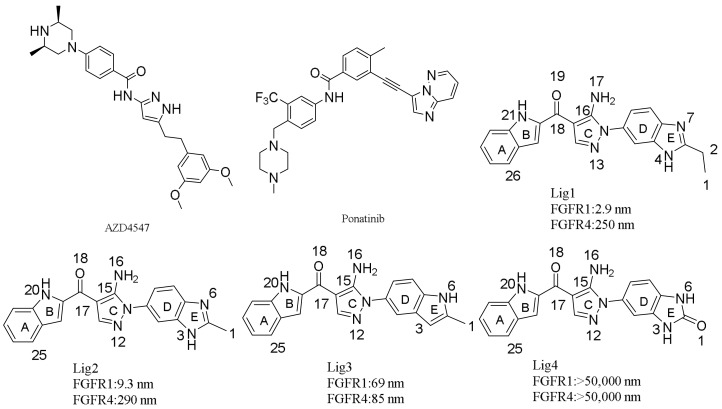
The structures of some FGFR inhibitors.

Since FGFRs play a meaningful role in tumors progression, some tyrosine-kinase inhibitors (TKIs) have been discovered to treat FGFR-dependent tumors [21]. In 2012, ponatinib (AP24543, Figure 1), developed by ARIAD Pharmaceuticals to be a FGFR inhibitor, was approved to be an oral drug for the treatment of chronic myeloid leukemia (CML) in the USA [22]. However, ponatinib didn’t show good enough selectivity to a typical FGFR isoform (FGFR1 IC_50_ = 2.23 nm; FGFR2 IC_50_ = 1.6 nm; FGFR3 IC_50_ = 18.2 nm; FGFR4 IC_50_ = 7.7 nm) [23]. Meanwhile, AZD4547 (Figure 1) is a novel skeleton FGFR inhibitor, which is in phase II clinical trials (FGFR1 IC_50_ = 0.2 nm; FGFR2 IC_50_ = 2.5 nm; FGFR3 IC_50_ = 1.8 nm; FGFR4 IC_50_ = 165 nm) [24]. In comparison with ponatinib, AZD4547 displayed much higher FGFR1-3 activities than FGFR4 and little adverse effects in clinical trials studies [23]. 

Since there is high residue identity around the ligand-binding pockets of FGFR1 and FGFR4, only a few inhibitors were developed to display more than 30-fold FGFR1 selectivity over FGFR4 [25]. Obviously, this may guide the design of isoform-specific inhibitors by exploring molecular mechanism of inhibitors binding to FGFR1 and FGFR4, respectively, and dynamics of ligand-receptor interactions. Although the ‘gatekeeper’ residue Val561 of FGFR1 was underlined as a key determinant for selectivity [26,27], adequate studies of structural basis of inhibitor-receptor interactions is always required in the development of its typical inhibitor. Recently, 1-(1*H*-benzimidazol-5-yl)-5-aminopyrazole derivatives were developed to be highly potent inhibitors with more than 80-fold selectivity for FGFR1 over FGFR4 (Figure 1), providing a chance to understand selectivity mechanism of ligand for FGFR1 vs. FGFR4 by molecular simulations studies [28].

In the current manuscript, molecular modeling simulations were carried out to study the selectivity mechanism of 1-(1*H*-benzimidazol-5-yl)-5-aminopyrazoles binding to FGFR1 and FGFR4, respectively. Four 1-(1*H*-benzimidazol-5-yl)-5-aminopyrazoles, Lig1, Lig2, Lig3, and Lig4 (Figure 1) were chosen to conduct molecular simulations for studies of their interaction modes and binding mechanism on either FGFR1 or FGFR4. Among these four compounds, Lig1 and Lig2 showed high (86-fold) and moderate (30-fold) selectivity to FGFR1 over FGFR4, respectively [28], while Lig3 displayed equal bioactivity against FGFR1 and FGFR4 and Lig4 did not display bioactivity to either FGFR1 or FGFR4. These results motivated us to investigate the molecular basis of selectivity of 1-(1*H*-benzimidazol-5-yl)-5-aminopyrazole derivatives binding to FGFR1 or FGFR4 using a combined computational approach. Meanwhile, comparative analyses of wild-type vs. two *in silico* mutants at residues Tyr563 and Ser565, respectively, of FGFR1 were also performed to get deeper insight into the molecular mechanism. 

Molecular docking [29] is an extensively applied approach to predict the binding mode of a ligand in the active site of target protein. Since docking could not produce an accurate enough model, docking-simulated structures are always further processed by molecular dynamics (MD) to get insight into the interaction kinetics and selectivity mechanism of ligands binding to target protein. In order to examine reliability and to distinguish interaction modes of different inhibitors within the binding pocket of target protein, binding free energies and residues contributions to ligand binding were also predicted through MMGB(PB)SA calculations on the basis of molecular dynamic trajectory. In this study, we take advantage of a special combination of computational techniques, including molecular docking, binding site comparison, MESP mapping, and MD simulations, to elucidate molecular features for achieving selectivity based on analyses of specific feature-by-residue interactions inducing different affinities of four 1-(1*H*-benzimidazol-5-yl)-5-aminopyrazoles binding to FGFR1 and FGFR4, respectively. It was proposed that H-bond interactions with Glu562 and Ala564 would make the ligand into FGFR1’s binding pocket in the correct orientation. Meanwhile, the pharmacophoric features to have H-bond interaction with Asp641 and hydrophobic interaction with Val561 and Phe642 were also suggested to be crucial for the affinity and selectivity of FGFR1 inhibitor. It is expected that the obtained results may be useful to the development of novel FGFR1-selective inhibitors by the rational design.

## 2. Computational Details

### 2.1. Preparation of Initial Complex

The co-crystal structure of Lig2-FGFR1, downloaded from RCSB Protein Data Bank (PDB ID: 5B7V), was applied as the starting point for molecular simulations. The initial conformations of another three inhibitors, Lig1, Lig3 and Lig4, were prepared by modifying the structure of Lig2 with its co-crystal conformation in 5B7V using the Sketch module of SYBYL-X 1.3 [30]. The generated Lig1, Lig3 and Lig4 were then respectively superimposed on Lig2 in 5B7V to replace Lig2 for generating initial structures of Lig1-FGFR1, Lig3-FGFR1 and Lig4-FGFR1.

Because of high sequence homology between FGFR1 and FGFR4 (Figure S1), an initial structure model of Lig2-FGFR4 was first generated based on the crystal structure of FGFR4 (PDB ID: 5JKG) using SYBYL-X 1.3. At first, 5JKG was aligned on 5B7V by superimposing backbones of FGFR1 and FGFR4, and Lig2 withdrawn from 5B7V was then merged into the binding pocket of FGFR4 to get a starting point of Lig2-FGFR4. Similarly, initial structural models of other complexes, Lig1-FGFR4, Lig3-FGFR4, and Lig4-FGFR4 were also prepared by replacing Lig2 in Lig2-FGFR4 complex with above in silico generated Lig1, Lig3, and Lig4, respectively.

Since there are two different residues, Tyr563 and Ser565, around the binding pocket of FGFR1, aligned with Cys552 and Ala554 of FGFR4, two different FGFR1 mutant models were also produced through in silico mutations at positions of Tyr563 and Ser565, respectively, to study the roles of these two residues interacting with the inhibitor. By mutating investigated residue of 5B7V with desired amino acid, Lig2-mutant complex was constructed through biopolymer module of SYBYL-X 1.3. In current studies, two FGFR1 mutants, named as Y563C (FGFR1) and S565A (FGFR1), were subjected to run flexible docking and MD simulations for the examination of the roles of Tyr563 and Ser565 in Lig2 binding to FGFR1.

#### 2.1.1. Binding Site Comparison

In order to distinguish differences of the inhibitor binding pockets between FGFR1 and FGFR4, preliminary binding site analyses were carried out on the crystal structures of FGFR1 (5B7V) and FGFR4 (5JKG) applying the Analysis Protein module of SYBYL-X 1.3. The Analysis Protein module employs a grid method [31] to dissect the ligand-binding pocket of target protein into a collection of potential binding volumes. Binding site comparison and characterization were proceeded by taking advantage of grid which is parameterized to depict the drug-like molecule [32]. Both FGFR1 and FGFR4 applied a ‘DFG-in’ mode binding site [19]. The residues around the binding pocket were figured using a 3D orthogonal grid. The area with a largest cluster of grids would be supposed as a favorable site for the binding of drug-like molecules.

#### 2.1.2. Docking Simulations

FlexiDock method of SYBYL-X 1.3 is one of widely used flexible docking programs [33]. Based on the above-prepared initial structural models of each complex, flexible docking modeling was carried out to predict binding modes of each ligand with either FGFR1 or FGFR4 using the FlexiDock method. Before docking calculations, the active site was defined to include all residues within a 6.0 Å radius around the inhibitor in each of above generated complexes. During FlexiDock simulations, all single bonds of both ligand and side-chains of residues in the outlined binding pocket were setup to be rotatable, while protein’s backbone conformation was kept rigid. When the top twenty solutions attained after 100,000 steps, docking was terminated and only the energetically favorable conformations were analyzed. Based on the ligand orientation, one structure for each ligand-receptor complex was selected to be a docking model for following computation.

#### 2.1.3. Molecular Dynamics Simulations 

Each of docking-predicted models of complexes Lig1-FGFR1, Lig3-FGFR1, Lig4-FGFR1, Lig1-FGFR4, Lig2-FGFR4, Lig3-FGFR4, Lig4-FGFR4, Lig2-Y563C (FGFR1), and Lig2-S565A (FGFR1) was subjected to run MD simulations in a solvation system for further stabilization and refinement using AMBER14 software package [34]. MD simulations were also carried out on the co-crystal structure of Lig2-FGFR1 for its dynamic characteristics in the water solution and the binding free energy computation. Before MD simulations, protein was added with protons by utilizing LEaP program of AMBER14 [35], and each complex was then solvated using a TIP3P water [36] molecules box with a margin distance of 9 Å. The generated solvation system was then neutralized using counterion Cl^−^ or Na^+^ to exchange appropriate water molecules by LEaP protocol. After the protein was assigned with AMBER ff99SB [37], and the ligand with general AMBER force filed [38], each complex was first minimized with three individual steps to remove any steric conflict occurred during system setup. At first step of energetic minimization, added ions and water molecules were optimized quickly with both protein and ligand frozen. During next step of minimizations, side-chains of amino acids were relaxed with protein backbone restrained under a constraint force of 5.0 kcal·(mol·Å^2^)^−1^. Finally, the whole system was fully optimized without any restriction. Each step of minimizations was carried out with 2500 steps of steepest descent followed by 5000 steps of conjugate gradient method. After energy minimizations, the temperature of each system was gradually increased in a *NVT* ensemble from 0 to 300 K during 100 ps MD simulations. The system was performed through Langevin dynamics with a collision frequency of 1 ps^−1^ under the force constant of 10 kcal·(mol·Å^2^)^−1^. In order to maintain the system stability, nine additional steps of MD equilibrations were carried out at the temperature of 300 K. In the meantime, the restraint weights of the whole system were gradually descending through 10, 8, 5, 3, 2, 1, 0.5, 0.1, and 0 kcal·(mol·Å^2^)^−1^. After MD equilibrations, the whole system was subjected to do MD productions for 50 ns by applying *NPT* ensemble with the temperature of 300 K and pressure of 1 atm. To constrain all bonds containing hydrogen atoms, integration step along with SHAKE algorithm [39] was used during 2 fs. Moreover, based on Particle-Mesh-Ewald (PME) method [40], the electrostatic interactions were calculated and the cutoff was set as 10 Å to treat non-bonded interactions. Dynamic trajectories were stored at every 1 ps during equilibration runs and 2 ps during MD production runs. All analyses during the MD simulations were performed by using the CARNAL, ANAL and CPPTRAJ modules of AMBER14 software package.

#### 2.1.4. Binding Free Energy Calculations and Per-Residue Free Energy Decomposition Analyses

MM/PB(GB)SA [40] methods were applied for predicting binding free energies (Δ*G*_bind_) of each inhibitor binding to either FGFR1, its mutants, or FGFR4 on the basis of a total of 1000 snapshots averagely withdrawn from the last 2 ns MD trajectories. The computations of binding free energies have been described in detailed in terms of the total molecular mechanics energy (gas phase, Δ*E*_MM_), solvation free energy (Δ*G*_sol_), and entropy contributions (*T*Δ*S*) in our previous publication [41,42]. As indicated, Δ*E*_MM_ was separated as internal energies (Δ*E*_int_), vdW energies (Δ*E*_vdW_) and non-bonded electrostatic energies (Δ*E*_ele_), the solvation-free energies (Δ*G*_sol_) summed by polar solvation free energies (Δ*G*_ele,sol_) and non-polar solvation contributions Δ*G*_nonpol,sol_. Furthermore, the free energy decomposition program in AMBER14 was run to obtain significant insight towards contributions of crucial residues responsible for ligand-receptor binding. The ligand-receptor interaction term described in equation below is comprised by insightful components, including vdW (Δ*G*_vdW_), electrostatic (Δ*G*_ele_), polar (Δ*G*_ele,sol_), and non-polar (Δ*G*_nonpol,sol_) contributions.

#### 2.1.5. Molecular Electrostatic Potential (MESP) Analyses

Density functional theory (DFT) calculations were performed for molecular electrostatic potentials of all four ligands Lig1, Lig2, lig3, and Lig4, respectively, on the basis of the co-crystal or MD-simulated binding conformations using Gaussian 09 (Gaussian, Inc., Wallingford, CT, USA) [43]. Electronic structure of each compound was optimized using the B3LYP method with 6-31g(d,p) basis set [44,45] in an emulational aqueous environment with the CPCM model [46] to depict physiological environmental conditions. After calculations, MESP maps of each ligand were produced by overlaying iso-potential surface on the iso-electron density surface (0.0004 e au^−3^) using GaussView. The color-coded iso-maps provided an intuitive interface through the overall molecular size and positive or negative electrostatic potential distributions around molecular surface based on its bioactive conformation. For example, deep-red color characterizes the most negative potential region with highest electron density. Additionally, the molecular surface with colors of cyan, yellow and green represent the moderate range of reactivity.

## 3. Results and Discussion

### 3.1. Molecular Docking

In order to verify our flexible docking protocol, docking simulations were first performed on Lig2 in FGFR1 active site using SYBYL-X 1.3/FlexiDock (Tripos, St. Louis, MO, USA) approach. Superimposition of the docking model of Lig2-FGFR1 on 5B7V (Figure S2B) indicated that the docked conformation of Lig2 may align with its FGFR1-cocrystal conformation with a root mean square deviation (RMSD) less than 0.7 Å. Therefore, it may be proposed that our docking procedure would be reliable to simulate binding modes of other inhibitors in FGFR1.

Since molecular structures of Lig1, Lig3 and Lig4 are similar to the structure of Lig2, our flexible docking simulations indicated that these compounds would have similar binding conformations and interaction modes with FGFR1 (Figure S2A,C). As illustrated in Figure S2A,B, both high potent and selective FGFR1 inhibitors Lig1 and Lig2 may have H-bond interactions with the backbones of Glu562, Ala564 and the side-chain of Asp641 of FGFR1. In addition, the ring C of Lig1 or Lig2 (Figure 1) may have hydrophobic interaction with the residues Val492, Val561 and Leu630. Furthermore, either C3-ethyl of Lig1 or C2-methyl of Lig2 may have C-π interaction with the side-chain phenyl of Phe642. On the other hand, Lig3, which showed about 10-times lower FGFR1 bioactivity than Lig1 or Lig2 and no selectivity against FGFR4, would not have a H-bond interaction with the side-chain of Asp641 since missing N-H in ring E, not like Lig1 or Lig2. Therefore, N4-H of ring E in Lig1 would be critical to FGFR1 inhibitory activity of the ligand by forming H-bond interaction with the carboxyl group of Asp641. As for Lig4 showing no activity to both FGFR1 and FGFR4, docking simulations suggested that it would not only be absent of the H-bond interaction with Asp641 but also lack the hydrophobic interaction with Phe642. In fact, other studies suggested that the key hydrophobic and H-bond interactions would play dominant role in the binding of FGFR1-selective inhibitor [19]. Our docking simulations further confirmed that Asp641 and Phe642 are key residues for FGFR1 bioactive inhibitor.

Since no co-crystal structure was reported to neither Lig1, Lig2, Lig3 nor Lig4 binding to FGFR4, flexible docking simulations were conducted to propose interaction modes of these four ligands, respectively, binding to FGFR4 based on its crystal structure using SYBYL-X 1.3/FlexiDock approach. Since FGFR1 and FGFR4 possess inhibitor-binding pockets with highly sequential identity, the starting point for docking simulations was obtained by superimposing the crystal structure of FGFR4 and previously docking-simulated structure of Lig2-FGFR1. After running 100,000 steps, the top ranked binding mode of each ligand binding to FGFR4 were chosen through *CScore* values. In the meantime, the selected binding modes were graphically visualized by taking advantage of MOLCAD module of SYBYL-X 1.3 to examine inhibitor-receptor interactions and to check up if the docked inhibitor is in a suitable direction. By analyzing the docking-simulated interaction modes of each ligand binding to FGFR4, it was found that Lig1, Lig2, Lig3, and Lig4 might have their N-H group to form H-bond interactions with Glu551 and Ala553, respectively, of FGFR4. Similar to the findings described above for ligand-FGFR1, the ring C of each ligand would be also surrounded by hydrophobic residues including Val481, Val550 and Leu619. Typically, the most FGFR4-active inhibitor Lig3 would have its N6-H group to form an extra H-bond interaction with Glu520. On the other hand, Lig4, displaying no activity to FGFR4, would not make neither an H-bond interaction with Glu520 nor an aromatic interaction with the side chain phenyl of Phe631.

### 3.2. Comparison of Binding Sites of FGFR1 and FGFR4

The inhibitor selectivity may be affected by a subtle shape variation in the side chain of different residues around binding sites of homology proteins. By comparing the spatial differences of inhibitor binding pockets of FGFR1 and FGFR4, it could be clued to design and develop novel selective inhibitors for either FGFR1 or FGFR4. As above docking simulations results indicated, the binding pockets of FGFR1 and FGFR4 are quite similar.

**Figure 2:**
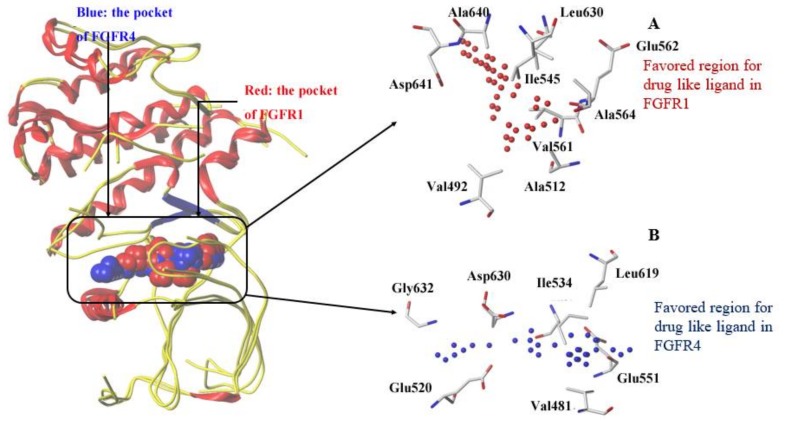
Comparison of the ligand binding site in X-ray structures of FGFR1 (red) and FGFR4 (blue). Colored grids indicate the strong interaction with corresponding residue: (**A**) top cluster of probe are shown as red spheres accumulated in a shallow hydrophobic pocket of FGFR1. The crucial residues constituting the binding site are shown as sticks; (**B**) top cluster of probe accumulated in a binding pocket of FGFR4 are shown as blue spheres. The crucial residues constituting the binding site are shown as sticks.

We made a comparison of binding pockets of FGFR1 and FGFR4 on the basis of their crystal structures using the Analysis Protein module of SYBYL-X 1.3. Grid method was applied for mapping the surface around the binding pocket of FGFR1 or FGFR4 in Analysis Protein module. Figure 2 depicts favorable areas for an inhibitor within the binding pockets of both FGFR1 and FGFR4.

Figure 2A displays the favorable region of FGFR1 for ligand is predominantly surrounded by residues Val492, Ala512, Ile545, Val561, Glu562, Ala564, Leu630, Ala640 and Asp641. These hydrophobic residues are actually around the ‘DFG-in’ binding site of FGFR1 to have good vdW and non-polar interactions with a potent inhibitor [18]. In FGFR4 (Figure 2B), the largest cluster region of grid is mainly composed of residues Val481, Glu520, Ile534, Glu551, Leu619, Asp630, and Gly632 to represent the favorable region for ligand binding. Binding site superimposition indicated that hydrophobic Tyr563 of FGFR1 was overlaid on hydrophilic Cys552 of FGFR4. Such a difference in the sizes of residues Cys and Tyr may rationalize the favorable accumulation of grid cluster in the corresponding area. As illustrated in Figure 1, each ligand contains a hydrophobic fused ring, and hydrophobic residues around the ligand could form hydrophobic interactions to enhance the affinity to FGFR1.

### 3.3. MESP Potential Surface Analyses

Mapping molecular electrostatic potentials (MESP) is a commonly applied protocol to characterize pharmacologically active molecules by analyzing electrostatic potential distributions, typically favorable to polar interactions, around molecular surface and frontier orbitals for chemical reactivity patterns. Generally, the areas of both negative and positive electrostatic potentials throughout the molecular surface may illustrate corresponding features to be an H-bond acceptor or donor for hydrophilic interactions with hydrophilic side-chain or backbone of residues, and different atomic charges may affect the H-bond strength. In comparison of different electrostatic distribution otherness on ligand’s surface and atomic charges, it is meaningful to understand molecular features influencing the binding affinity and selectivity of the ligand in different isoform proteins. In current work, our attention is paid to research electrostatic distribution throughout the molecular surface of the four ligands in Figure 1.

**Figure 3:**
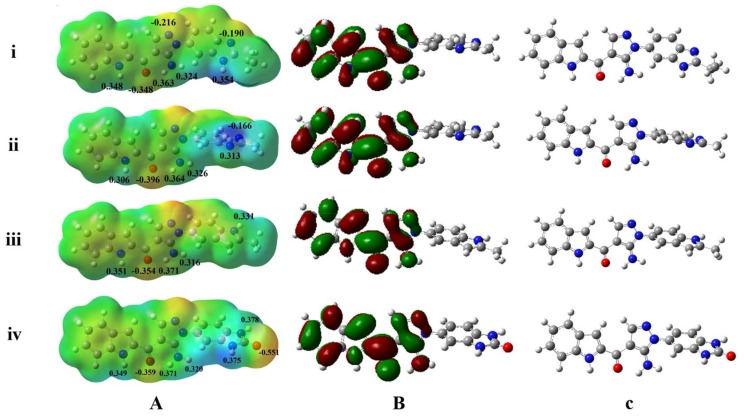
Comparison of the electrostatic potential and HOMO-LUMO orbitals of ligands for FGFR1 and FGFR4. (**A**) MESP superimposed onto a surface of constant density and Mulliken atomic charges mapped onto the compounds; (**B**) HOMOs and LUMOs orbitals with B3LYP/6-31g(d,p); (**C**) the structures of the compounds. The variation in MESP from positive to negative is shown in red to blue scale. (**i**–**iv**) The calculation results of four ligands (Lig1, Lig2, Lig3, and Lig4).

The MESP mappings reveal that FGFR1-selective inhibitors Lig1 and Lig2 share unique electronic properties (Figure 3a), which are different from Lig3 and Lig4 (Figure 3a). Both Lig1 and Lig2 have the most electronegative potential region (deep red color) locating around the carbonyl oxygen atom. In fact, the carbonyl oxygen atom of Lig1 and Lig2 bear average Mulliken charges of −0.348 and −0.396, respectively, displaying molecular region to have H-bond interaction with a H-bond donor from residues. The electronegative potential region would have high coulomb interaction with electropositive potential region of target protein or have H-bond interaction with –NH or –OH group of side-chain or backbone of residues. Another negative region was found to be located around the N atom in the 1*H*-benzimidazole moiety of Lig1 or Lig2 with Mulliken charges of −0.190 and −0.166, respectively. On the other hand, the N4-H located on the 1*H*-benzimidazole moiety of Lig1 displays higher positive Mulliken charge than N3-H of Lig2 (Lig1: 0.354; Lig2: 0.313). It indicated that Lig1 would have stronger H-bond interaction between N4-H group and Asp641 of FGFR1 than Lig2 as shown in above discussion of docking simulation results. This difference could be a factor for Lig1 showing a little higher bioactivity and selectivity to FGFR1 than Lig2.

In comparison with Lig1 or Lig2, Lig3, which showed similar bioactivities towards FGFR1 (69 nM) and FGFR4 (85 nM), possesses similar charge distributions on rings A, B and C, and may also form the H-bond interactions with key residues Glu562 and Ala564 of FGFR1. On the other hand, Lig3 lacks a positive charge on benzimidazole like Lig1 or Lig2, which could make Lig3 not to possess an H-bond interaction with Asp641 of FGFR1. Such a finding could be a reason for Lig3 to show near 10 times lower bioactivity to FGFR1 than Lig1 or Lig2. Nevertheless, it was suggested that such a positive charge would be necessary for an inhibitor strongly binding to FGFR1 by comparing FGFR1 bioactivities of above three inhibitors. Lig4, without good binding affinities to both FGFR1 and FGFR4, displays the unique charge distributions. Its most negative charge (deep red color) region is located around the O1 atom (Mulliken charge: −0.551) of ring E. At this position of molecular structure, Lig1 or Lig2 contains a hydrophobic group, which was proposed to form C-π interaction with residue Phe642 of FGFR1 or residue Phe631 of FGFR4 by co-crystal structure or docking simulations. Therefore, such hydrophobic interactions would be critical for ligand binding to FGFR1 or FGFR4.

### 3.4. HOMO and LUMO Orbitals Analyses

The frontier HOMO and LUMO orbitals were computed for all four inhibitors Lig1, Lig2, Lig3, and Lig4 using the quantum chemical software Gaussian 09. As reported [42], the LUMO value may reflect the electron acceptor ability of a ligand in complex formation with its receptor, and the electron donating ability of the inhibitor could be directly associated with its HOMO value. Figure 3b displays both HOMO and LUMO orbitals plotted onto the molecular surface along with the HOMO and LUMO energy gaps of Lig1, Lig2, Lig3 and Lig4. The calculated results indicate that both Lig1 and Lig2 are a little more reactive than Lig3 and Lig4, because of the rapid electron transfer ability of Lig1 and Lig2 (Figure S3). In addition, the most FGFR1-activity compound Lig1 shows the highest dipole moment (7.472 Debye) than another three inhibitors (Table S2). As we performed, the ab initio calculations were carried out for each ligand in its binding conformation to get their MESP, atomic charges, and LUMO and HOMO values in an aqueous environment with the CPCM model but not in the state with target protein. Our studies ligands were reversible inhibitors without covalently bonding with FGFR, their electronic structures would not be influenced by protein residues. Therefore, above discussed electronic properties would not be changed observably in complex with FGFRs.

### 3.5. Analyses of Structure Stability and Flexibility

**Figure 4:**
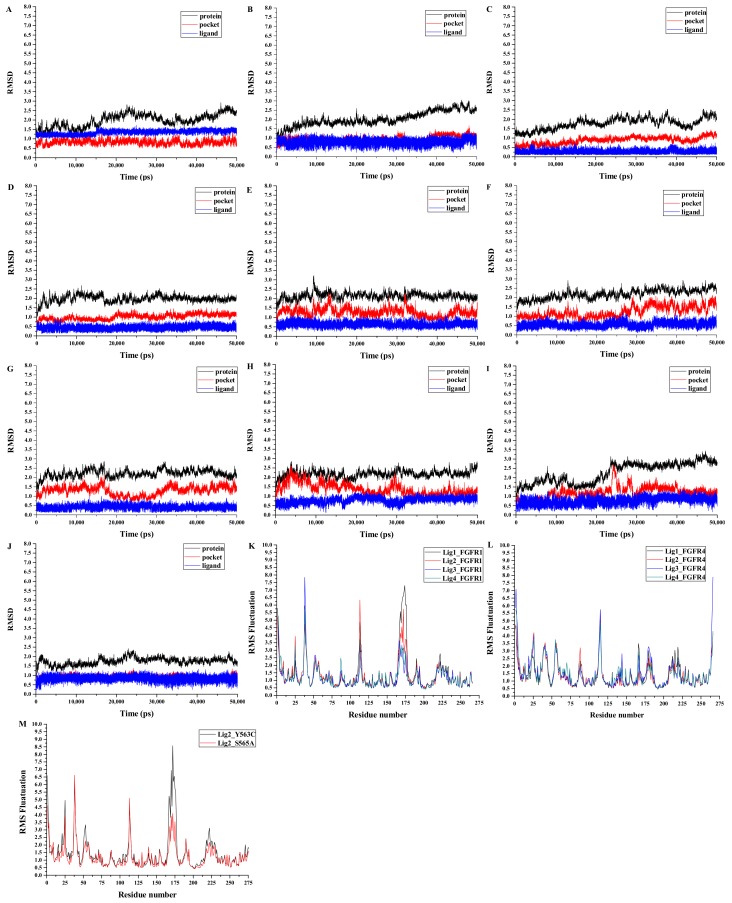
RMSDs of Cα atoms of the protein, backbone atoms of binding pocket (within 5.0 Å), and the heavy atoms in the ligand for: (**A**) Lig1-FGFR1; (**B**) Lig2-FGFR1; (**C**) Lig3-FGFR1; (**D**) Lig4-FGFR1; (**E**) Lig1-FGFR4; (**F**) Lig2-FGFR4; (**G**) Lig3-FGFR4; (**H**) Lig4-FGFR4; (**I**) Lig2-Y563C (FGFR1); (**J**) Lig2-S565A (FGFR1); (**K**) RMSF of residues for four FGFR1 complexes obtained from 50 ns MD simulation; (**L**) RMSF of residues for four FGFR4 complexes obtained from 50 ns MD simulation; (**M**) RMSF of residues for two FGFR1-mutant complexes obtained from 50 ns MD simulation.

MD simulations were carried out for 50 ns production run in the explicit water environment for each of ten systems, including four FGFR1 complexes Lig1-FGFR1, Lig2-FGFR1, Lig3-FGFR1, and Lig4-FGFR1, two FGFR1 mutant complexes Lig2-Y563C (FGFR1) and Lig2-S565A (FGFR1), and four FGFR4 complexes Lig1-FGFR4, Lig2-FGFR4, Lig3-FGFR4 and Lig4-FGFR4. In order to check the dynamic stability of protein with bound ligand during MD productions, root-mean-square deviation (RMSD) fluctuations of both protein and inhibitor were checked for every system using CPPTRAJ module in AMBER14. Figure 4 displays RMSD curves for whole protein, residues around binding pocket, and ligand, respectively, of each system for entire snapshots saved along 50 ns MD trajectories in relative to its starting structure. Since it was detected that each complex arrived at equilibrium after initial fluctuations, it was rational to conduct calculations of binding free energies and energy decomposition on the basis of the conformations withdrawn from MD simulations using MMPB(GB)SA methods.

In addition, the root-mean-square fluctuation (RMSF) was also computed for each MD-simulated system. As shown in Figure 4, similar RMSFs were observed for all systems (FGFR1, FGFR4 and FGFR1-mutant), indicating similar binding features for all inhibitors on target proteins. Among all ten systems, Lig2-Y563C (FGFR1) complex was found to have the highest RMSF fluctuations among all ten MD-simulated systems, suggesting unstable binding of Lig2 in FGFR1-mutant Y563C (FGFR1). Since Lig2 is a highly potent and selective FGFR1 inhibitor, it may be signified that FGFR1’s Tyr563 plays an important role in Lig2 binding to FGFR1. Meanwhile, as shown in Figure 4M, the largest RMSF section is near the binding pocket. In this section, it contains the crucial residues, including Val561, Glu562, Cys563, Leu630, Asp641 and Phe642, for an inhibitor binding to FGFR1. It indicates that the mutation Tyr563 to Cys563 would affect residues spatial orientation around the ligand and further make the binding conformation unstable. In fact, Tyr563 was found to possess some parallel π-π interaction with ring A in the co-crystal structure of Lig2-FGFR1.

### 3.6. Binding Free Energy Calculations

To verify the inhibitor binding strengths in FGFR1 and FGFR4, respectively, and to certify the residue contributions to inhibitor’s binding, MM/PBSA and MM/GBSA calculations were performed to predict binding free energies and corresponding energy decomposition over 1000 snapshots extracted from last 2 ns of MD trajectories for each of ten complexes. It was summarized of calculated binding free energies in terms of gas phase energy, solvation energy, and entropic contributions for ten complexes in Table 1. Additionally, it was also listed of binding free energies derived from experimental IC_50_ values of four inhibitors on either FGFR1 or FGFR4. Although the calculated binding affinity values were not absolutely equal to IC_50_-determined values, the variation tendency of Δ*G*_pred(GB)_ values of inhibitors Lig1, Lig2, Lig3, and Lig4 (−20.38 ± 1.92, −14.70 ± 1.94, −12.08 ± 3.57, −9.67 ± 3.70 kcal/mol, respectively) binding to FGFR1 fitting together well with the changing trends of their experimental IC_50_ values (FGFR1: Lig1, IC_50_ = 2.9 nm; Lig2, IC_50_ = 9.3 nm; Lig3, IC_50_ = 69 nm; Lig4, IC_50_ > 50 μM). Moreover, calculated Δ*G*_pred(GB)_ value of Lig1 or Lig2, binding to FGFR1 is also lower than corresponding Δ*G*_pred(GB)_ for FGFR4, respectively. This result indicated that both Lig1 and Lig2 bind more stably and tightly with FGFR1 than FGFR4. On the other hand, because of Lig1 possessing the highest selectivity to FGFR1 among four inhibitors, the Δ*G*_pred(GB)_ values difference of Lig1 to FGFR1 and FGFR4 is more significant than another three ligands, Lig2, Lig3 and Lig4.

As indicated in the superimposition of the crystal structures of Lig2-FGFR1 and FGFR4 (Figure 2), Tyr563 and Ser565 of FGFR1’s binding pocket are two residues different from corresponding residues (Cys552 and Ala554) of FGFR4’s binding pocket, we also performed the binding free energy calculations for structure models of Lig2 with two in silico FGFR1 mutants, respectively. As listed in Table 1, the calculated Δ*G*_pred(GB)_ of Lig2-FGFR1 is more negative than that of either Lig2-Y563C (FGFR1) or Lig2-S565A (FGFR1), indicating the important roles of these two residues to interact with Lig2. In the meantime, it may also be suggested of these two residues playing positive effect in FGFR1 bioactivity and selectivity against FGFR4. In fact, Lig2 was observed to form some parallel π-π interaction with residue Tyr563 of FGFR1 in the co-crystal structure of Lig2-FGFR1, indicating the crucial role of Tyr563 for a potent inhibitor of FGFR1.

To apprehend the binding process of each complex in detail, the total binding free energy was further decomposed into individual components. As shown in Figure S4, vdW and non-polar interactions were predicted to be the most important contributors for each ligand binding to either FGFR1 or FGFR4, and the sum of vdW energy (Δ*E*_vdW_) and non-polar solvation energy (Δ*G*_nonpolar,sol_) is a favorable contributor to each inhibitor binding to FGFR1 and FGFR4. Moreover, vdW interactions would be noticeable differences of Lig1, Lig2, Lig3, and Lig4 binding to FGFR1, FGFR1 mutants, and FGFR4. This finding is rational since there are a few hydrophobic residues, like Leu484 (Leu473), Ile545 (Ile534), Val561 (Val550), Ala564 (Ala553), Gly567 (Gly556), and Leu630 (Leu619), within 5 Å around each of inhibitors in the binding pocket of FGFR1 (FGFR4). It was also noticed that the difference of vdW energy components between Lig1-FGFR1 and Lig1-FGFR4 (9.05 kcal/mol) is much higher than that between Lig3-FGFR1 and Lig3-FGFR4 (2.49 kcal/mol).

In comparison with Lig1 and Lig2, it could be suggested that the decrease of vdW contributions may be responsible for the reduction of FGFR1 bioactivity of Lig2. Actually, C3-ethyl of Lig1 could form stronger C-π interaction with Phe642 of FGFR1 than C2-methyl of Lig2 based on the docking results described above. In addition, electrostatic energies have obvious differences either in the predicted binding free energies of different inhibitors binding to FGFR1 or FGFR4. Interestingly, Lig1 displayed the highest bioactivity and selectivity to FGFR1 versus FGFR4, but Lig1 was predicted to have lower electrostatic interactions with FGFR1 than FGFR4. Therefore, vdW interactions could be main determinants for bioactivity and selectivity of inhibitors Lig1 and Lig2 to FGFR1 versus FGFR4 although electrostatic interactions may make contributions to the binding affinity of an inhibitor. Analyzing energetic components suggested that the decrease of FGFR1 bioactivity would be directly associated with the reduction in vdW and non-polar energy contribution. On the other hand, the electrostatic energy (Δ*E*_ele_) component contribution of Lig1-FGFR1, Lig2-FGFR1, Lig3-FGFR1, and Lig4-FGFR1 gradually increased. Hence, the different binding affinities of each inhibitor (Lig1, Lig2, Lig3, and Lig4) in FGFR1 versus FGFR4 could be decided by vdW and non-polar solvation free energies, while the electrostatic components make important influence either to FGFR1 binding affinity and selectivity over FGFR4.

### 3.7. Dynamic Analyses of the Selectivity Mechanism of Inhibitors

**Figure 5:**
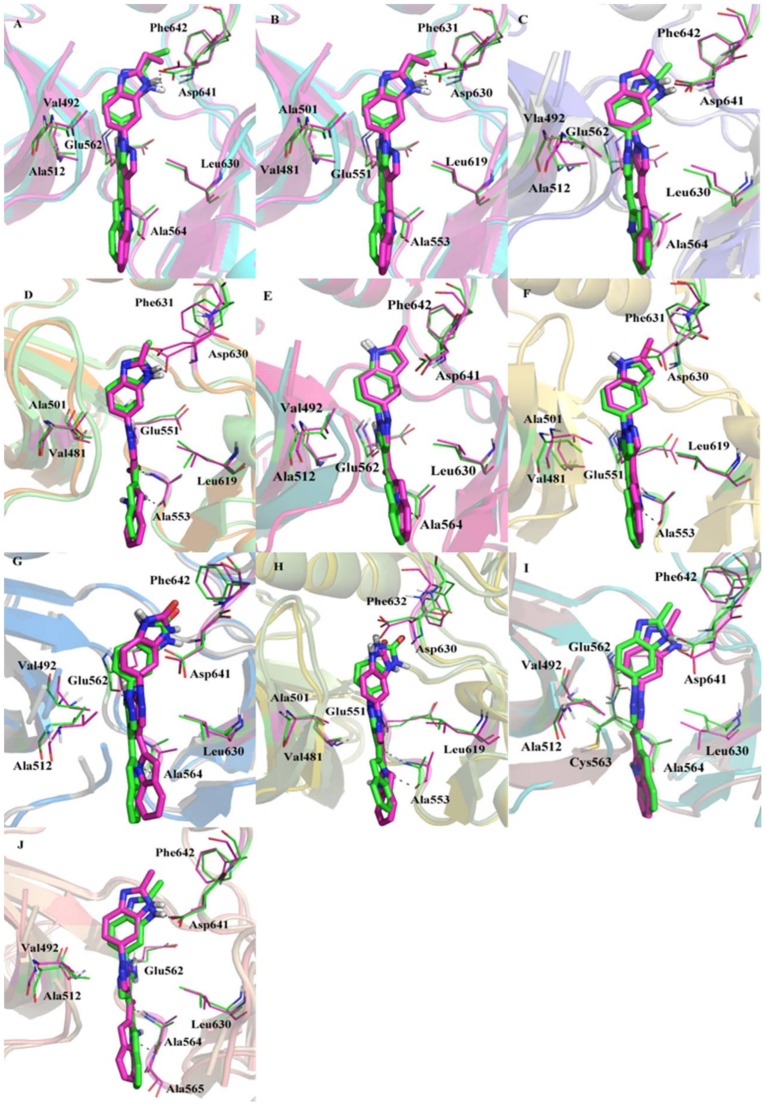
Structure comparison between initial (green) and representative snapshots from MD (magenta) of: (**A**) Lig1-FGFR1; (**B**) Lig1-FGFR4; (**C**) Lig2-FGFR1; (**D**) Lig2-FGFR4; (**E**) Lig3-FGFR1; (**F**) Lig3-FGFR4; (**G**) Lig4-FGFR1; (**H**) Lig4-FGFR4; (**I**) Lig2-Y563C (FGFR1); (**J**) Lig2-S565A (FGFR1). Line: binding pocket; stick: ligand.

In order to observe the conformational stability of an inhibitor in the binding pockets of FGFR1 and FGFR4, a representative MD-simulated structure of each ligand-FGFR1/FGFR4 complex was superimposed onto the corresponding starting structure (Figure 5). The structure comparison indicated that each inhibitor may possess the similar interaction modes with FGFR1 and FGFR4. The MD results indicated that the N-H group of indole ring and carbonyl group of every inhibitor could form a typical H-bond network with the backbone of Ala564 of FGFR1 or Ala553 of FGFR4. In addition, the N-H group in ring C of four inhibitors also could form the H-bond interaction with the backbone oxygen of either FGFR1 Glu562 or FGFR4 Glu551. Furthermore, both N4-H of Lig1 and N3-H of Lig2 may form another specific H-bond interaction with the side-chain of highly conserved residue Asp641 of FGFR1 (Figure 5A) or Asp630 of FGFR4 (Figure 5B).

**Figure 6:**
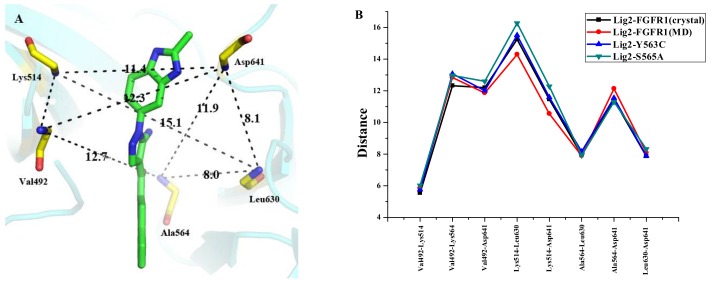
(**A**) The active site of FGFR1 (residues with backbone atoms). The black dash represent the key atoms to define the frame of active site; (**B**) The distances between the atoms in backbone at the key positions.

Additionally, it was revealed that in silico mutation of either Y563C or S565A of FGFR1 would not affect the binding conformation of Lig2 in FGFR1 mutant by comparing MD simulated structural models of Lig2-FGFR1 and Lig2-Y563C (FGFR1) or Lig2-S565A (FGFR1) (Figure 5I and Figure 6J). In order to interpret the extent of crucial residues spatial variation and geometric change in binding site produced by site mutation, the active site frame was depicted from the backbone nitrogen atom of residues Val492, Lys514, Ala564, Leu630, and Asp641, which surround the ligand in the binding site of FGFR1 (Figure 6A). Furthermore, it was measured of the distances of the backbone nitrogen atoms of these residues in FGFR1-mutant and FGFR1. As illustrated in Figure 6B, the site mutation is adjacent to the residues forming FGFR1 active pocket, indicating both Tyr563 and Ser565 play meaningful roles in the correct geometrical frame in the active site.

### 3.8. Insight to the Activity Difference of Lig1 and Lig4 to FGFR1

**Figure 7:**
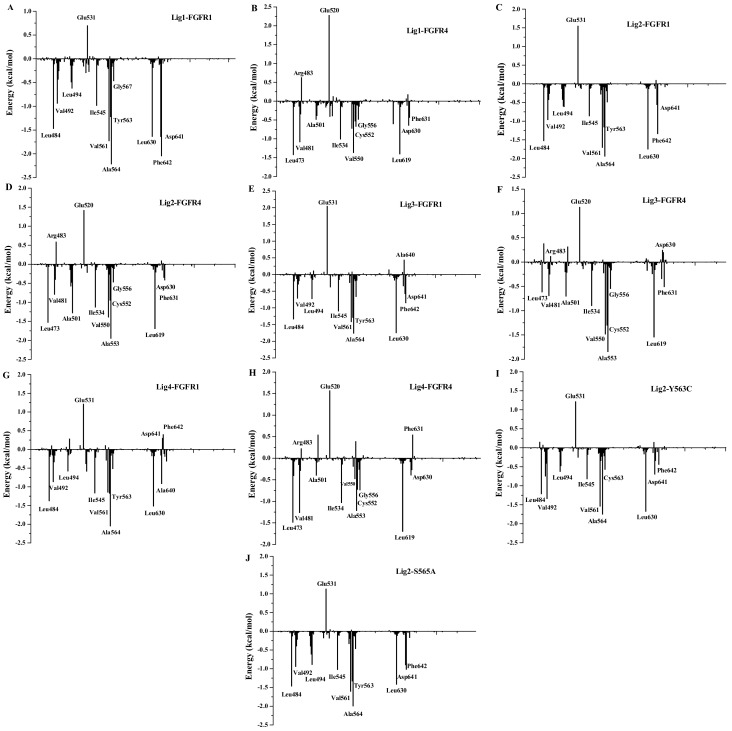
The interaction spectra of inhibitor-residue: (**A**) Lig1-FGFR1; (**B**) Lig1-FGFR4; (**C**) Lig2-FGFR1; (**D**) Lig2-FGFR4; (**E**) Lig3-FGFR1; (**F**) Lig3-FGFR4; (**G**) Lig4-FGFR1; (**H**) Lig4-FGFR4; (**I**) Lig2-Y563C (FGFR1); (**J**) Lig2-S565A (FGFR1).

To illustrate the binding mechanism of Lig1 and Lig4 showing different activity and selectivity to FGFR1, the predicted binding free energy of each complex was further decomposed to be residue contributions. Figure 7 illustrates averaged interaction energies decomposed to individual residue contributions based on their electrostatic and vdW components for whole system. Meanwhile, the comparison of vdW and non-polar energy with electrostatic and polar energy was also performed (Figure S5). By comparing residue contributions to Lig1 (Figure 7A) or Lig4 (Figure 7G), it was found that FGFR1 would have its residues Leu484, Val492, Val493, Leu494, Ile545, Val561, Tyr563, Ala564, Gly567 and Leu630 to favorably interact with Lig1 or Lig4.

There are acidic residue Asp641 and hydrophobic residue Phe642 to have favorable electrostatic and vdW interactions with Lig1, respectively, but not with Lig4. As discussed above, Asp641 may have an H-bond interaction with N4-H group in benzimidazole of Lig1. Such an H-bond would have 99% occupancy during the MD simulations by H-bond analysis using the CPPTRAJ module of Amber14 [47] (Table S3). However, as displayed in Table S3, Asp641 may only have 15% occupancy of H-bond interaction with Lig4. Therefore, it is rational for Asp641 to have −1.64 kcal/mol calculated contribution to Lig1 binding to FGFR1 (Figure 7A) but 0.38 kcal/mol to Lig4 (Figure 7G). Phe642 is an aromatic residue next to Asp641, its side-chain phenyl ring would be pointed by an ethyl group substituted on benzimidazole ring to have C-π interaction with each other. The oxygen atom of carbonyl group at the corresponding site of Lig4 would not have a suitable interaction with the side-chain of Phe642. As a result, Phe642 would have −2.05 kcal/mol calculated contributions to Lig1 binding to FGFR1 (Figure 7A) but unfavorable 0.46 kcal/mol to Lig4 (Figure 7G).

However, Glu531 was observed to have unfavorable contributions to FGFR1 binding of either Lig1 (0.72 kcal/mol) and Lig4 (1.24 kcal/mol). In fact, the co-crystal structure of Lig2-FGFR1 illustrated that Glu531 was close to N6 of Lig2 to have a negative contribution to Lig2’s binding neither (Figure 7C). Therefore, it was suggested that an H-bond donor would be necessary to have favorable interactions with Glu531 for the purpose of the increase of FGFR1 bioactivity of the inhibitor.

### 3.9. Insight to the Activity and Selectivity of Lig1 in Complexes Lig1-FGFR1 and Lig1-FGFR4

Energy-decomposed results may be used to investigate the individual residue contribution to a ligand tightly binding in the active site of protein. As described above, we have figured out the residues to have favorable contributions for Lig1 binding to FGFR1. Since FGFR1 and FGFR4 are two high homology proteins (Figure S1), there are many conserved residues, like Leu473, Val481, Val482, Ile534, Val550, Ala553, Gly556, Leu619, and Asp630, to make the major favorable energy contribution for Lig1 binding to FGFR4 (Figure 7B). Typically, FGFR4 may have two residues Ala553 and Asp630, aligned to FGFR1’s Ala564 (−2.22 kcal/mol) and Asp641 (−1.64 kcal/mol), respectively, to show lower favorable contributions of −0.67 kcal/mol and −0.68 kcal/mol to Lig1 binding to FGFR4 (Figure S6). Actually, CPPTRAJ H-bond analyses indicated that H-bond distance between O19 of Lig1 and N-H of FGFR4’s Ala553 would be 2.38 Å, which is a little longer than that between O19 of Lig1 and N-H of FGFR1’s Ala564 (2.18 Å). Moreover, it was also predicted of unstable H-bond formed between N4-H of Lig1 and carboxyl O atom of FGFR4’s Asp630, with an occupancy of 27% during MD simulations. As described above, there are two different residues, Cys552 and Ala554, around the binding pocket of FGFR4, in alignment with Tyr563 and Ser565 of FGFR1. Tyr563 of FGFR1 would have some parallel π-π interaction to make favorably energetic contribution of −1.24 kcal/mol for Lig1 binding to FGFR1, but Cys552 of FGFR4 only have energetic contribution of −0.49 kcal/mol for Lig1 binding to FGFR4. As illustrated in Figure 7B, there would be another two residues, Arg483 and Glu520 of FGFR4, to have unfavorable electrostatic interactions with Lig1. Lig1-FGFR4 was counteracted by negative electrostatic interactions. 

Additionally, Figure S6A revealed that the C3-ethyl group of Lig1 would form a strong hydrophobic interaction with Phe642 (−2.05 kcal/mol) of FGFR1 with an energetic contribution much more preferable to Lig1’s binding in FGFR1 than the corresponding residue Phe631 (−0.62 kcal/mol) of FGFR4. Moreover, computational simulations suggested that ‘gatekeeper’ residue Val561 (−1.73 kcal/mol) of FGFR1 may form strong hydrophobic interaction with Lig1 to contribute energetically more than the aligned Val548 (−0.62 kcal/mol) of FGFR4 to Lig1’s binding. As displayed in Figure S6A, residues Tyr563 and Leu630 may also make more positive contributions of vdW and non-polar solvation energies to FGFR1 than the corresponding residues Cys552 and Leu619 of FGFR4, respectively. The difference in vdW and non-polar solvation energies were responsible for the different affinity between Lig1-FGFR1 and Lig1-FGFR4 complexes. In Figure S6B, the total electrostatic energies of residues Ala564 (Ala553 of FGFR4) and Asp641 (Asp630 of FGFR4) of FGFR1 also yield the different energetic difference between Lig1-FGFR1 and Lig1-FGFR4 complexes. All the data discussed above would illustrate a selectivity mechanism of Lig1 binding to FGFR1 over FGFR4.

### 3.10. Insight to the Activity and Selectivity of Lig2 in Complexes Lig2-FGFR1, Lig2-Mutants, and Lig2-FGFR4

As illustrated in Figure 7C, FGFR1’s residues Leu484, Val492, Leu494, Ile545, Val561, Tyr563, Ala564, Leu630, Asp641, and Phe642 would have favorable energetic contributions for Lig2 binding to FGFR1. Meanwhile, the residues that favorably contribute to Lig2 binding in FGFR4 include Leu473, Val481, Ala501, Ile534, Val550, Cys552, Ala553, Gly556, Leu619, Asp630, and Phe631. On the other hand, Glu531 (1.62 kcal/mol) would form unfavorable electrostatic energy contributions to Lig2 binding to FGFR1, which is similar in Lig1-FGFR1 complex. Like Lig1, Arg483 of FGFR4 would not have favorable interactions with Lig2 neither. In fact, MD simulations indicated that hydrophobic rings A and B of Lig2 push the hydrophilic side-chain of Arg483 away from the active site of FGFR4.

The comparison of the different Δ*G*_ligand-residues_s for Lig2 binding to either FGFR1 or FGFR4 was performed (Figure S7) to rationalize the higher affinity of Lig2 towards FGFR1 than FGFR4. As a result, three residues Val561 (Val548), Tyr563 (Cys552) and Phe642 (Phe631) would be identified as main determinants for selectivity of Lig2 binding to FGFR1 and FGFR4, respectively, with big differences of vdW and non-polar energetic contributions. All these three residues would have hydrophobic interactions with the corresponding features of Lig2. Typically, Typ563 of FGFR1 would have energetic contribution of −2.25 kcal/mol to Lig2. In comparison, the aligned residue Cys552 of FGFR4 would contribute −1.25 kcal/mol energetic interaction with Lig2. In fact, the co-crystal structure of Lig2-FGFR1 revealed that the aromatic side-chain of Tyr563, located at the beginning of FGFR1’s binding pocket, have some parallel π-π interaction with Lig2’s fused-ring of A and B, while Cys552 of FGFR4 would certainly have a lower energetic C-π interaction. Besides, in silico mutagenesis analyses were carried out to further confirm the individual role of typical residues in the ligand-receptor interaction. In comparison of Lig2-FGFR1 and Lig2-Y563C (FGFR1) complexes, the mutation of Tyr563 to Cys563 would induce the binding free energy decline by 5.46 kcal/mol (Table 1). Similarly, it is also energetically unfavorable for Lig2 binding to FGFR1 mutant S565A. Furthermore, the mutation at Tyr563 was found to be unfavorable for vdW and electrostatic energy components (Δ*E*_vdW_ = 3.86 kcal/mol; Δ*E*_ele_ = 5.39 kcal/mol) compared with Lig2-FGFR1 complex (Table 1). When Tyr563 mutates to Cys552 which is a hydrophilic residue and has smaller volume, it would lead to the spatial conformation of binding pocket subtle change, and further affect the ligand binding mode. As revealed in Figure S8A, the vdW and non-polar energy of Tyr563 (−2.3 kcal/mol) in Lig2-FGFR1 is much higher than Cys563 (−1.20 kcal/mol) in Lig2-Y563C (FGFR1).

In addition, the C2-methyl group of Lig2 is closer to the center of Phe642 (3.24 Å) of FGFR1 than to the center of the aligned residue Phe631 (4.25 Å) of FGFR4. The data could explain the reason why Phe642 (−1.14 kcal/mol) of FGFR1 may have higher vdW and non-polar interactions with Lig2 than Phe631 (−0.21 kcal/mol) of FGFR4. Moreover, the ‘gatekeeper’ residue Val561 (−1.76 kcal/mol) of FGFR1 also may have higher vdW and non-polar energy contribution to Lig2’s binding than the corresponding residue Val548 (−0.30 kcal/mol) of FGFR4. 

The energy decomposition analyses also suggested that it would be the acidic residue Asp641 (−0.36 kcal/mol) of FGFR1 but not the aligned residue Asp630 (0.10 kcal/mol) of FGFR4 to make favorable electrostatic energy contributions for Lig2’s binding. Our MD simulations indicated that Asp641 of FGFR1 would have its O atom to make more stable H-bond interaction, 85% occupancy listed in Table S3, with N3-H of Lig2 than aligned residue Asp630, 32% occupancy, of FGFR4. All the results discussed above will explain the mechanism of Lig2 selectivity towards FGFR1 over FGFR4.

### 3.11. Insight to the Bioactivity and Selectivity of Lig1 and Lig3 Binding to FGFR1 in Complexes of Lig1-FGFR1 and Lig3-FGFR1

Among the four compounds, Lig3 display almost equal bioactivities to FGFR1 (IC_50_ = 69 nm) and FGFR4 (IC_50_ = 85 nm). As listed in Table 1, the calculated binding free energies of Lig3-FGFR1 (Δ*G*_pred(PB)_ = −10.19 kcal/mol and Δ*G*_pred(PB)_ = −12.08 kcal/mol) are higher than those of both Lig1-FGFR1 (Lig1 IC_50_ (FGFR1) = 2.9 nm, Δ*G*_pred(PB)_ = −22.03 kcal/mol and Δ*G*_pred(PB)_ = −20.38 kcal/mol) and Lig2-FGFR1 (Lig2 IC_50_ = 9.3 nm, Δ*G*_pred(PB)_ = −15.50 kcal/mol and Δ*G*_pred(PB)_ = −14.70 kcal/mol) and lower than those of Lig4-FGFR1 (Lig4 IC_50_ > 50 μM, Δ*G*_pred(PB)_ = −7.38 kcal/mol and Δ*G*_pred(GB)_ = −9.67 kcal/mol), while the calculated binding free energies of Lig3-FGFR4 (Δ*G*_pred(PB)_ = −15.71 kcal/mol and Δ*G*_pred(PB)_ = −17.71 kcal/mol) are lowest among four complexes because of its highest FGFR4 bioactivity. As we discussed above, the binding free energy calculations would have rational results although the predicted binding free energies did not match the experimental ones derived from IC_50_s.

The binding free energies of Lig3-FGFR1 and Lig3-FGFR4 complexes were further decomposed to per-residue contributions to rationalize the affinity of Lig3 towards FGFR1 or FGFR4. As illustrated in Figure S8E,F, residues Leu484, Val492, Leu494, Ile545, Val561, Tyr563, Ala564, Leu630, Asp641 and Phe642 of FGFR1 are favorable for Lig3 binding to FGFR1, and the residues Leu473, Val481, Ala501, Ile534, Val550, Cys552, Ala553, Gly556, Leu619 and Phe631 of FGFR4 positively contribute to Lig3’s binding.

In Lig3-FGFR1 complex, the hydrophilic residue Glu531 is also unfavorable for Lig3 binding to FGFR1 like Lig1 or Lig2. As for Lig3-FGFR4 complex, the unfavorable interaction with Arg483 and Glu520 were also found. In fact, in comparison with the docking and MD simulation results of Lig1-FGFR4 and Lig3-FGFR4, the N6-H of Lig3 could form weak hydrophilic interaction with Glu520 (Figure S9B), while the N7 group of Lig1 is without this interaction. Therefore, it may be a reason for Lig3 showing better affinity to FGFR4 than Lig1 or Lig2. Moreover, the Mulliken charge of N6-H of Lig3 is 0.331 which is favorable for negative charge attack (Figure 3). On the contrary, the Mulliken charge of Lig1’s N7 is −0.190, which gets away from negative charge. Hence, if we add some H-bond donor groups, like –OH, at N6 of Lig3 to make more stable and tight H-bond interaction with Glu520 of FGFR4, it may be helpful for the enhancement the bioactivity and selectivity of Ligand binding to FGFR4.

By comparing crucial-residues energy decompositions for Lig1-FGFR1 with Lig3-FGFR1 (Figures S5 and S8), it was found that Val561, Asp641, and Phe642 of FGFR1 would have more positively contributions to Lig1’s binding in FGFR1 than Lig3’s binding in FGFR1 (Val561, −1.68 kcal/mol for Lig1 vs. −1.38 kcal/mol for Lig3; Asp641, −1.64 kcal/mol for Lig1 vs. −0.51 kcal/mol for Lig3; and Phe642, −2.05 kcal/mol for Lig1 vs. −0.82 kcal/mol for Lig3). Further analyses suggested that the big difference of binding free enerygies betwee Lig1-FGFR1 and Lig3-FGFR1 would be mainly raised from the electrostatic energy contributions of Asp641 with −1.3 kcal/mol in Lig1-FGFR1 and 0.32 kcal/mol in Lig3-FGFR1. As described above, Lig1 may have its N3-H group to form a stable H-bond interaction (>99% occupancy listed in Table S3) with Asp641 but Lig3 would miss such an H-bond with Asp641. However, Lig3 would miss such an H-bond with Asp641 (0% occupancy in Table S3) since its H-bond donor N6-H did not have suitable orientation to point on Asp641. Phe642 is another critical FGFR1 residue to have a little big difference for Lig3 binding to FGFR1 in comparison with Lig1. That would be because Lig3 has a shorter C2-CH_3_ group of Lig3 to make a lower favorable vdW and non-polar interactions with Phe642 (−1.13 kcal/mol) than Lig1 (−2.17 kcal/mol) with a C2-ethyl group. The hydrophobic interaction with Phe642.

## 4. Conclusions

In this work an atomic perspective concerning the selectivity of four ligands Lig1, Lig2, Lig3, and Lig4 towards FGFR1 against FGFR4 is examined. Especially, we characterized potential interactions that have major affects in inhibitors binding to FGFR1 or FGFR4. By applying a series of computational techniques, the bioactivity and selectivity mechanism of four inhibitors was studied for binding to FGFR1 or FGFR4. Based on the Analysis Protein module of SYBYL-X 1.3, the binding site surface comparison of these two high homology isoforms was performed. MESP was carried out to figure out the role of negative and positive charges in determining bioactivity and selectivity to FGFR1 over FGFR4. The N-H group in benzimidazole of Lig1 or Lig2 forms the strong and stable H-bond interaction with Asp641, and this interaction induces the hydrophobic group close to the residue Phe642, which leads to form strong hydrophobic interaction. In comparison with Lig1 and Lig3, Lig3 lacks H-bond interaction with Asp641 and enough hydrophobic interaction with Phe642. Additionally, between Lig1 or Lig2 and Lig4, Lig4 lacks the strong and stable H-bond interaction with Asp641 and hydrophobic interaction with Phe642, and it makes Lig4 not reveal the bioactivity to FGFR1. Therefore, among the three inhibitors Lig1, Lig2 and Lig3, the H-bond interaction with the backbone of residue FGFR1’s Ala564 induces the ligand forming the proper direction into the binding pocket, and the H-bond interaction with Asp641 and hydrophobic interaction with Phe642 enhance the activity and selectivity to FGFR1. Furthermore, the ‘gatekeeper’ residue Val561 and Tyr563 also play a positive role in the bioactivity and selectivity to FGFR1. The functional and structural information obtained from the present work are hope to help the designing of novel selective compounds.

## Figures and Tables

**Table 1:** Binding free energies and components of ten studied systems ^a^.

Complex System	Lig1-FGFR1	Lig2-FGFR1	Lig3-FGFR1	Lig4-FGFR1	Lig1-FGFR4	Lig2-FGFR4	Lig3-FGFR4	Lig4-FGFR4	Lig2-Y563C ^d^	Lig2-S565A ^d^
Δ*E*_vdW_	−54.86 ± 2.42	−45.82 ± 2.53	−44.76 ± 2.45	−41.53 ± 2.67	−45.81 ± 2.65	−41.76 ± 2.77	−42.27 ± 2.87	−39.21 ± 2.94	−41.96 ± 2.57	−43.04 ± 2.75
Δ*E*_ele_	−15.61 ± 2.64	−22.93 ± 2.82	−24.96 ± 3.09	−28.95 ± 4.67	−24.57 ± 3.48	−27.92 ± 5.18	−25.32 ± 3.90	−20.66 ± 9.64	−17.54 ± 3.02	−23.01 ± 2.80
Δ*G*_nonpol,sol_	−4.21 ± 0.10	−4.16 ± 0.09	−4.10 ± 0.10	−3.96 ± 0.09	−4.32 ± 0.11	−4.19 ± 0.10	−4.29 ± 0.11	−4.01 ± 0.13	−4.20 ± 0.10	−4.08 ± 0.09
Δ*G*_ele,sol(PB)_	30.58 ± 3.03	37.40 ± 3.26	38.10 ± 3.12	38.81 ± 3.08	40.78 ± 4.87	40.80 ± 5.02	37.01 ± 4.59	31.98 ± 7.94	31.53 ± 3.59	34.16 ± 2.58
Δ*G*_ele,sol(GB)_	32.23 ± 2.99	38.20 ± 2.99	36.21 ± 2.56	36.52 ± 2.60	37.41 ± 2.93	39.87 ± 4.39	31.34 ± 3.21	32.27 ± 7.67	31.31 ± 2.36	36.30 ± 2.52
Δ*E*_vdW_ + Δ*G*_nonpol,sol_	−59.07 ± 2.52	−49.98 ± 2.62	−48.86 ± 2.55	−45.49 ± 2.76	−50.13 ± 2.76	−45.95 ± 2.87	−46.56 ± 2.98	−43.22 ± 3.07	−46.16 ± 2.67	−47.12 ± 2.84
Δ*E*_ele_ + Δ*G*_ele,sol(PB)_	14.97 ± 5.67	14.47 ± 6.08	13.14 ± 6.21	9.86 ± 7.75	16.21 ± 6.35	12.88 ± 6.42	11.69 ± 6.31	11.32 ± 7.89	13.99 ± 6.61	11.15 ± 5.38
Δ*E*_ele_ + Δ*G*_ele,sol(GB)_	16.62 ± 5.63	15.27 ± 5.81	7.25 ± 5.65	7.57 ± 7.27	12.84 ± 6.41	11.95 ± 5.47	8.02 ± 5.71	11.61 ± 7.51	13.77 ± 5.38	13.29 ± 5.32
*T*Δ*S*	−22.07 ± 6.23	−20.01 ± 6.49	−25.53 ± 3.63	−28.25 ± 6.33	−21.39 ± 4.63	−21.86 ± 5.42	−22.83 ± 5.67	−26.66 ± 8.09	−23.15 ± 7.17	−23.86 ± 5.74
Δ*G*_pred(PB)_	−22.03 ± 1.96	−15.50 ± 2.21	−10.19 ± 4.13	−7.38 ± 3.18	−12.53 ± 3.48	−11.21 ± 3.87	−15.71 ± 3.62	−5.24 ± 2.87	−9.02 ± 2.11	−12.11 ± 2.48
Δ*G*_pred(GB)_	−20.38 ± 1.92	−14.70 ± 1.94	−12.08 ± 3.57	−9.67 ± 3.70	−15.9 ± 3.54	−12.14 ± 2.92	−17.71 ± 3.02	−4.95 ± 2.49	−9.24 ± 0.88	−9.97 ± 2.42
IC_50 (nM)_^b^	2.9	9.3	69	>50,000	250	290	85	>50,000	NA	NA
Δ*G*_exp_^c^	−11.71	−11.02	−9.82	NA	−9.06	−8.97	−9.70	NA	NA	NA

^a^ All energies are in kcal·mol^−1^; the MM-PB(GB)SA calculated binding free energy Δ*G*_pred_ = Δ*E*_vdW_ + Δ*G*_nonpol,sol_ + Δ*E*_ele_ + Δ*G*_ele,sol_ − *T*Δ*S*; ^b^ The IC_50_ values of Lig1, Lig2, Lig3, and Lig4 were taken from reference; ^c^ The experimental binding free energy Δ*G*_exp_ ≈ −*RT* lnIC_50_; ^d^ Two mutant Lig2-FGFR1 complexes.

## Supplementary Materials

The following are available online.
