# Supplementary material for: Computational Simulation Studies on the Binding Selectivity of 1-(1H-Benzimidazol-5-yl)-5-aminopyrazoles in Complexes with FGFR1 and FGFR4
Source: Molecules. 2018 Mar 1;23(4):767. doi: 10.3390/molecules23040767 (PMC6017917; doi:10.3390/molecules23040767)
Supplement: Supplementary file 1 [file molecules-23-00767-s001.pdf]

Supplementary Material

Computational Simulation Studies on the Binding Selectivity of 1-(1*H*-Benzimidazol-5-yl)-5-aminopyrazoles in Complexes with FGFR1 and FGFR4

You-Lu Pan, Yan-Ling Liu and Jian-Zhong Chen *

College of Pharmaceutical Sciences, Zhejiang University, Hangzhou 310058, P.R.China; 11319001@zju.edu.cn (Y.-L.P.); liuyanling610@zju.edu.cn (Y.-L.L.)

* Correspondence: chjz@zju.edu.cn; Tel./Fax: +86-571-88208659

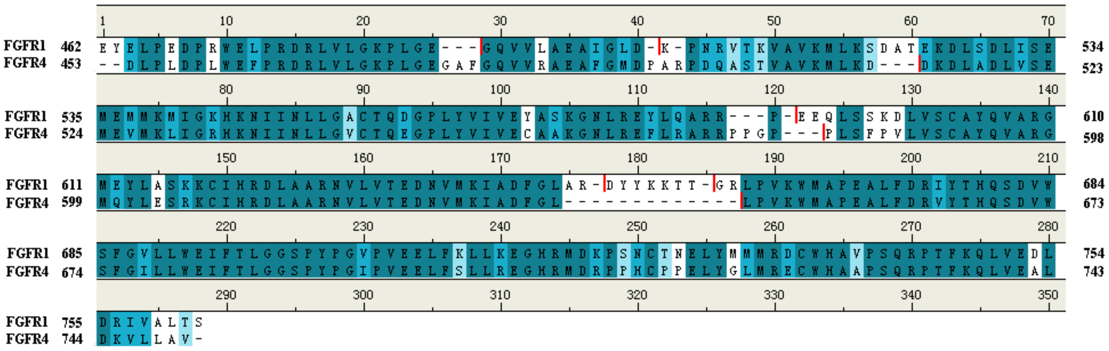

Figure S1. Amino acid sequence alignment of FGFR1/FGFR4

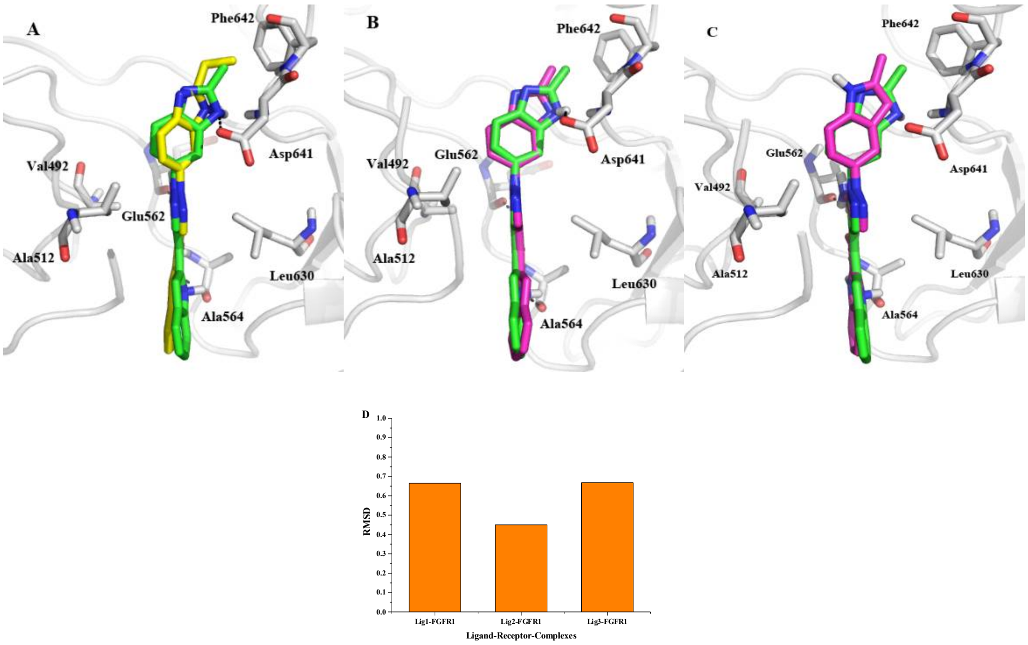

Figure S2. Comparison of binding modes of docked ligand with their starting conformation. (A) Superimposition of docked ligand Lig1 (Yellow) and initial conformation of FGFR1 (Green). (B) Superimposition of docked ligand Lig2 (Purple) and experimental conformation of FGFR1 (Green). (C) Superimposition of docked ligand Lig3 (Purple) and initial conformation of FGFR1 (Green). (D) The average means RMSD using bars chat.

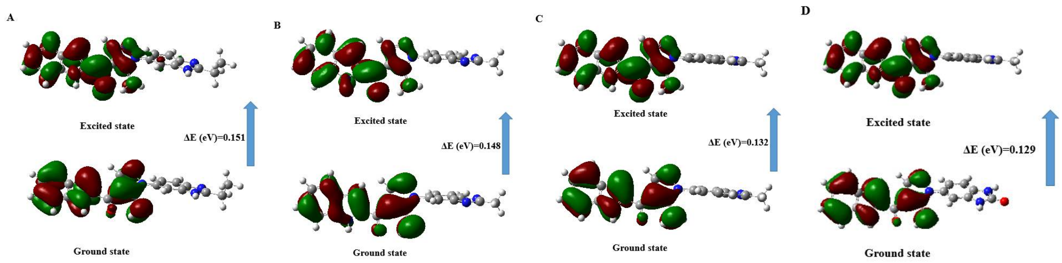

Figure S3. Molecular orbital for the HOMO-LUMO plot of (**A**) Lig1, (**B**) Lig2, (**C**) Lig3, and (**D**) Lig4 with B3LYP/6-31G(d,p).

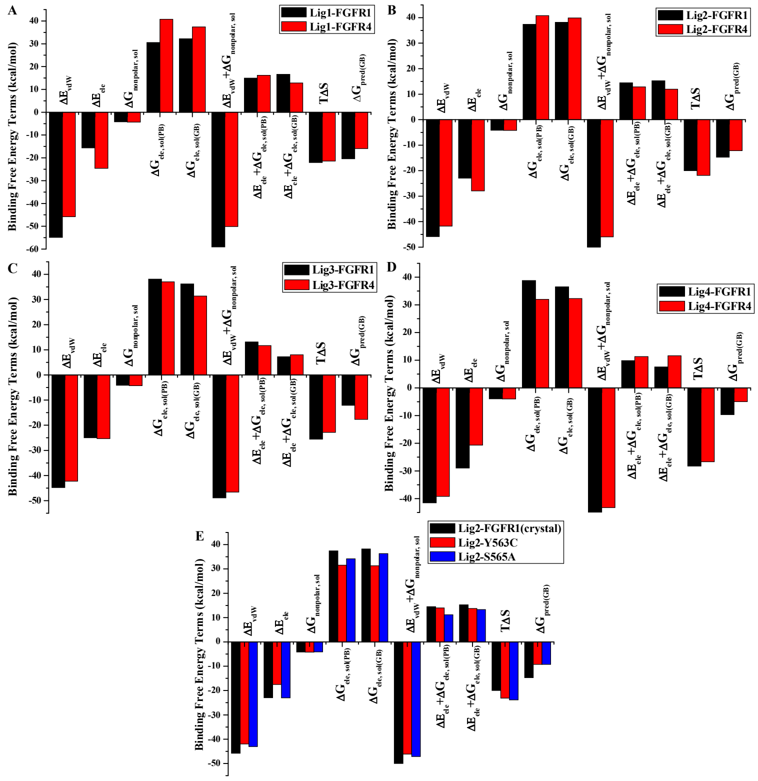

Figure S4. Comparison between binding free energy terms of FGFR1 and FGFR4: (**A**) Lig1, (**B**) Lig2, (**C**) Lig3, (**D**) Lig4, (**E**) Lig2-FGFR1-mutant.

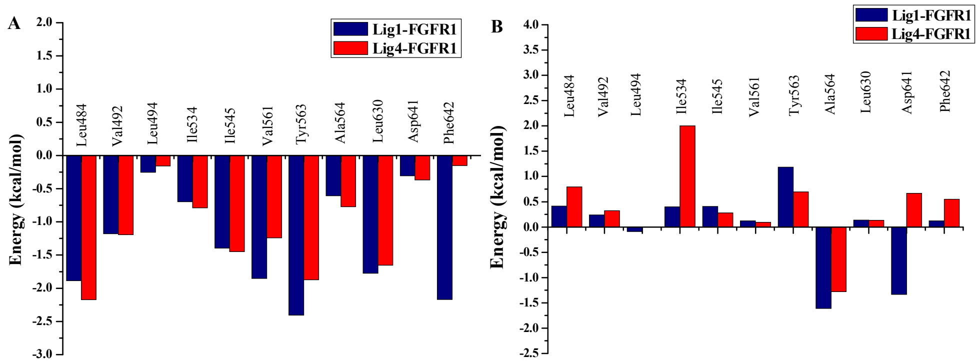

Figure S5. The comparison of crucial-residues energy decomposition for Lig1-FGFR1 system: (A) the sums of vdW and nonpolar solvation ($\Delta G_{vdW}+\Delta G_{nonpolar,sol}$) of Lig1-FGFR1 (Blue) and Lig4-FGFR1 (Red), (B) the sums of electrostatic and polar energy ($\Delta G_{ele}+\Delta G_{ele,sol}$) of Lig1-FGFR1 (Blue) and Lig4-FGFR1 (Red).

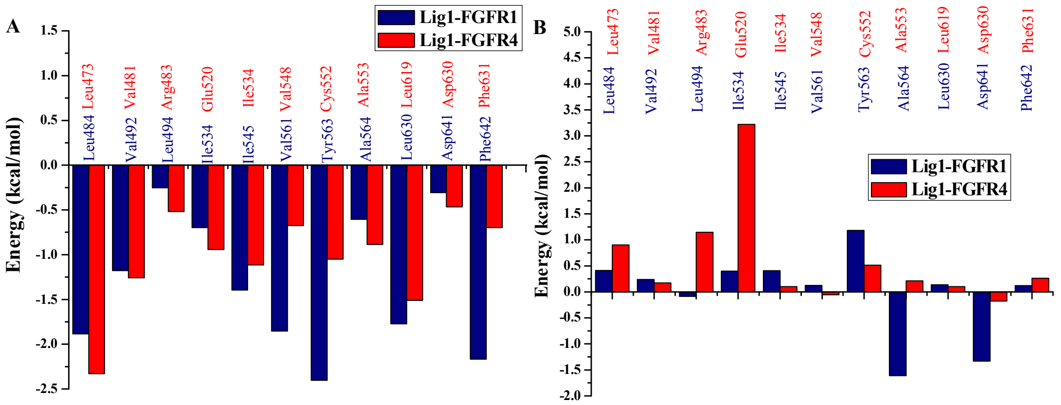

Figure S6. The comparison of crucial-residues energy decomposition for Lig1-FGFR1 system: (A) the sums of vdW and nonpolar solvation ($\Delta G_{\text{vdW}}+\Delta G_{\text{nonpolar,sol}}$) of Lig1-FGFR1 (Blue) and Lig1-FGFR4 (Red), (B) the sums of electrostatic and polar energy ($\Delta G_{\text{ele}}+\Delta G_{\text{ele,sol}}$) of Lig1-FGFR1 (Blue) and Lig1-FGFR4 (Red).

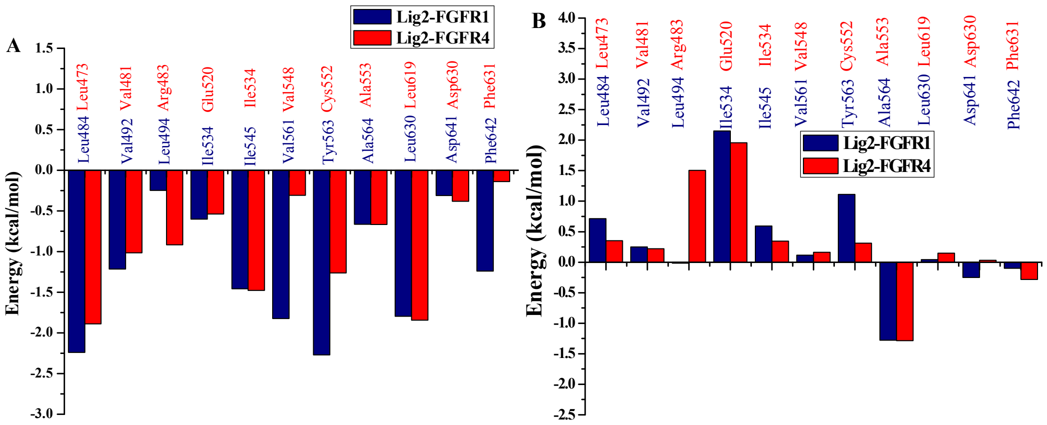

Figure S7. The comparison of crucial-residues energy decomposition for Lig2-FGFR1 system: (A) the sums of vdW and nonpolar solvation ($\Delta G_{\text{vdW}}+\Delta G_{\text{nonpolar, sol}}$) of Lig2-FGFR1 (Blue) and Lig2-FGFR4 (Red), (B) the sums of electrostatic and polar energy ($\Delta G_{\text{ele}}+\Delta G_{\text{ele, sol}}$) of Lig2-FGFR1 (Blue) and Lig2-FGFR4 (Red).

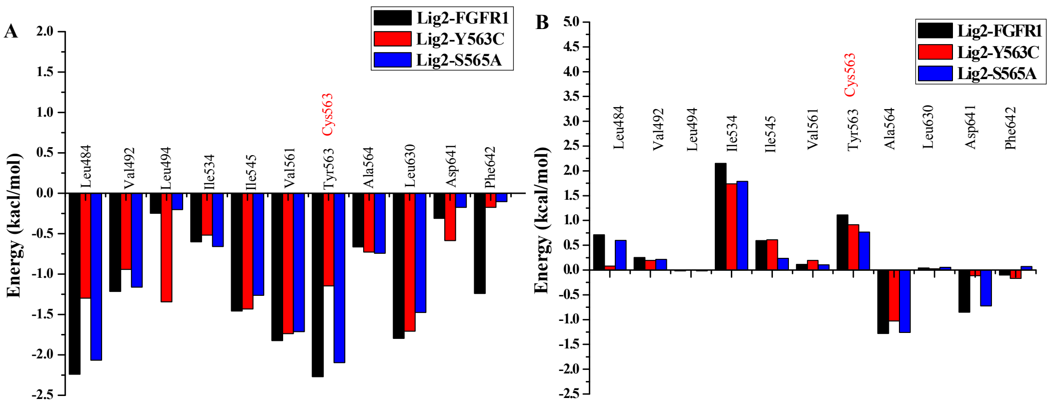

Figure S8. The comparison of crucial-residues energy decomposition for Lig2-FGFR1 system: (A) the sums of vdW and nonpolar solvation (ΔG_{vdW}+$\Delta G_{nonpolar,\ sol}$) of Lig2-FGFR1 (Black), Lig2-Y563C (Red) and Lig2-S565A (Blue); (B) the sums of electrostatic and polar energy (ΔG_{ele}+$\Delta G_{ele,\ sol}$) of Lig2-FGFR1 (Black), Lig2-Y563C (FGFR1) (Red) and Lig2-S565A (FGFR1) (Blue).

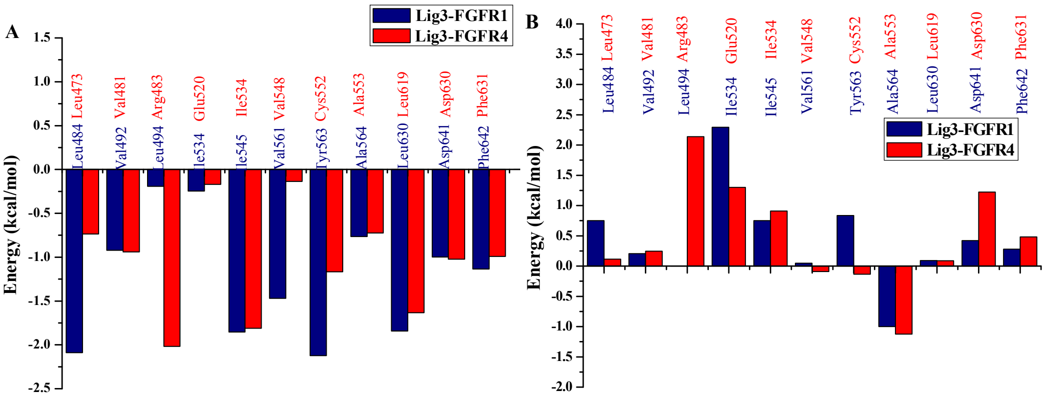

Figure S9. The comparison of crucial-residues energy decomposition for Lig3-FGFR1 system: (A) the sums of vdW and nonpolar solvation (ΔG_{vdW}+$\Delta G_{nonpolar,\ sol}$) of Lig3-FGFR1 (Blue) and Lig3-FGFR4 (Red), (B) the sums of electrostatic and polar energy (ΔG_{ele}+$\Delta G_{ele,\ sol}$) of Lig3-FGFR1 (Blue) and Lig3-FGFR4 (Red).

Table S1. Surflex score of docked ligand Lig1-2 for FGFR1 and Lig1 for FGFR4

**Docking complex**	ΔG_{exp}	**CScore**[a]	**Crash score**[b]	**Polar score**[c]	**G score**[d]	**PMF score**[e]	**D score**[f]	**Chem score**[g]
Lig1-FGFR1	-14.67	7.56	-1.05	2.17	-265.89	-59.87	-148.28	-29.89
Lig2-FGFR1	-10.43	6.50	-1.05	1.89	-234.12	-32.98	-124.67	-24.59
Lig1-FGFR4	-8.27	4.25	-1.10	1.66	-189.72	-23.64	-108.54	-21.67

[a]**CScore** is a consensus scoring which uses multiple types of scoring functions to rank the affinity of ligands. [b]**Crash-csore** revealing the inappropriate penetration into the binding site, [c]**Polar** region of the ligand, [d]G-score showing hydrogen bonding, complex (ligand-protein), and internal (ligand-ligand) energies, [e]**PMF-score** indicating the Helmholtz free energies of interactions for protein-ligand atom pairs (Potential of Mean Force, PMF), [f]**D-score** for charge and van der Waals interactions between the protein and the ligand, [g]**Chem-score** points for hydrogen bonding, lipophilic contact, and rotational entropy, along with an intercept term.

Table S2. Quantum chemical descriptors based upon DFT calculations used for MESP for compounds Lig1, Lig2, Lig3, and Lig4.

Quantum descriptors	Lig1	Lig2	Lig3	Lig4
E_{LUMO} (eV)	-0.068	-0.074	-0.086	-0.090
E_{HOMO} (eV)	-0.219	-0.222	-0.218	-0.219
Total dipole moment μ（D）	7.472	6.236	6.547	2.190

Table S3. H-bonds analysis from MD [a] .

System	Donor	Acceptor	Occupancy(%)[b]	Distance(Å)[c]	Angle(°)[d]
Lig1-FGFR1	Ala564 N-H	Lig1 O19	100	2.18±0.2	164°
	Ala564 O	Lig1 N21 H	100	3.12±0.12	154°
	Asp641 O	Lig1 N4 H	100	2.64±0.23	143°
	Glu562 O	Lig1 N17 H	75	3.25±0.13	132°
Lig2-FGFR1	Ala564 N-H	Lig2 O18	100	2.24±0.15	165°
	Ala564 O	Lig2 N20 H	100	3.10±0.20	155°
	Asp641 O	Lig2 N3 H	85	3.65±0.10	135°
	Glu562 O	Lig2 N16 H	76	3.64±0.15	135°
Lig3-FGFR1	Ala564 N-H	Lig3 O18	95	2.25±0.43	162°
	Ala564 O	Lig3 N20 H	96	3.32±0.54	154°
	Glu562 O	Lig3 N16 H	54	3.54±0.76	137°
Lig4-FGFR1	Ala564 N-H	Lig4 O18	56	3.10±0.75	152°
	Ala564 O	Lig4 N20 H	56	3.89±0.34	145°
	Asp641 O	Lig4 N6 H	15	3.78±0.21	132°
	Glu562 O	Lig4 N16 H	56	3.33±0.56	132°
Lig1-FGFR4	Ala553 N-H	Lig1 O19	100	2.38±0.2	165°
	Ala553 O	Lig1 N21 H	100	3.12±0.12	154°
	Asp630 O	Lig1 N4 H	27	3.64±0.13	130°
	Glu551 O	Lig1 N17 H	75	3.15±0.13	133°
Lig2-FGFR4	Ala553 N-H	Lig2 O18	100	2.34±0.58	169°
	Ala553 O	Lig2 N20 H	100	3.51±0.20	150°
	Asp630 O	Lig2 N3 H	32	3.78±0.87	131°
	Glu551 O	Lig2 N16 H	67	3.64±0.15	135°
Lig3-FGFR4	Ala553 N-H	Lig3 O18	100	2.05±0.48	169°
	Ala553 O	Lig3 N20 H	100	3.68±0.78	167°
	Glu551 O	Lig3 N16 H	65	3.89±0.56	137°
Lig4-FGFR4	Ala553 N-H	Lig4 O18	70	3.71±0.32	142°
	Ala553 O	Lig4 N20 H	68	3.12±0.38	135°
	Asp630 O	Lig4 N6 H	34	3.98±0.29	139°
	Glu551 O	Lig4 N16 H	51	3.53±0.86	123°
Lig2-Y563C	Ala564 N-H	Lig2 O18	100	2.84±0.27	134°
	Ala564 O	Lig2 N20 H	100	3.60±0.76	127°
	Asp641 O	Lig2 N3 H	68	4.12±0.10	147°
	Glu562 O	Lig2 N16 H	78	3.67±0.57	138°
Lig2-S565A	Ala564 N-H	Lig2 O18	100	3.84±0.27	138°
	Ala564 O	Lig2 N20 H	100	3.70±0.16	147°
	Asp641 O	Lig2 N3 H	85	3.66±0.47	150°
	Glu562 O	Lig2 N16 H	61	3.13±0.78	149°

[a] The listed donor and acceptor pairs satisfy the criteria (H-bond length less than 5 Å and H-bond angle (120°-180°) for the hydrogen bond over 30.0% of the time during the 50 ns of MD simulation. [b] Occupancy is the percentage of H-bond formed during the investigated time period. [c] The average distance with standard error (SE=stand deviation/$N^{1/2}$) between H-bond acceptor and proton on H-bond donor in the investigated time period. [d] The average H-bond angle with standard error (SE=standard deviation/$N^{1/2}$) in the investigated time period.
